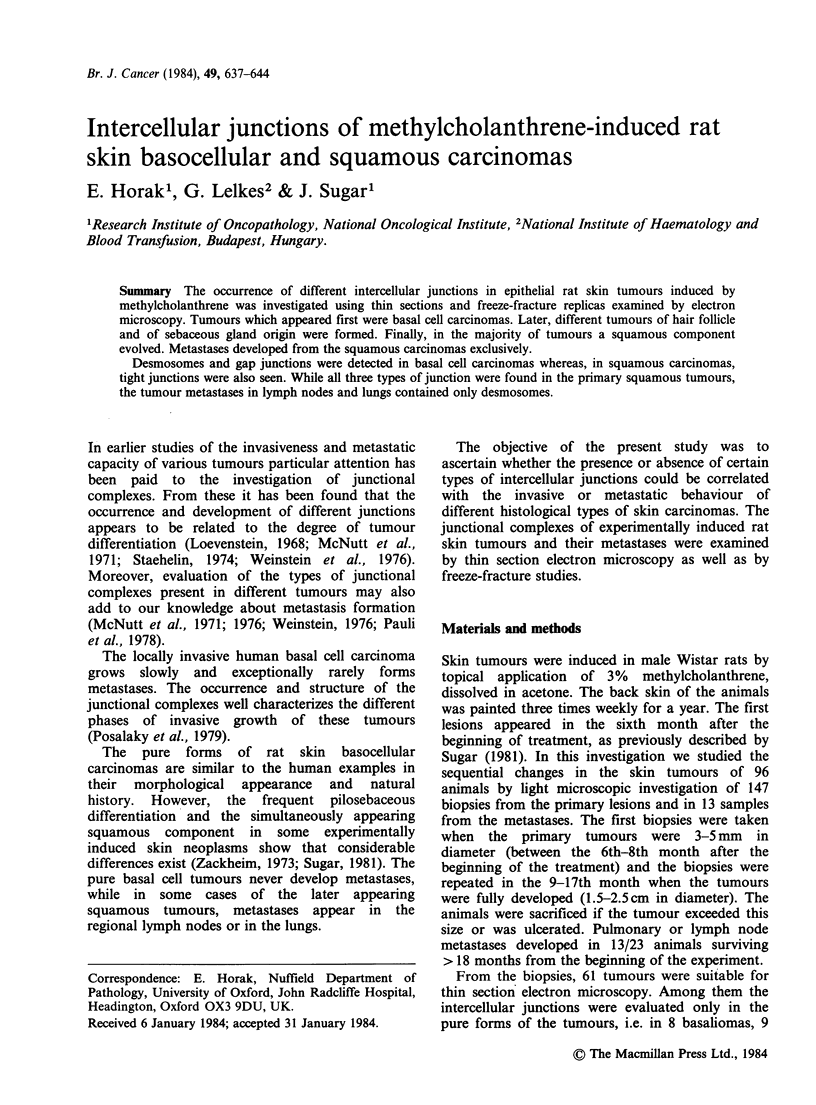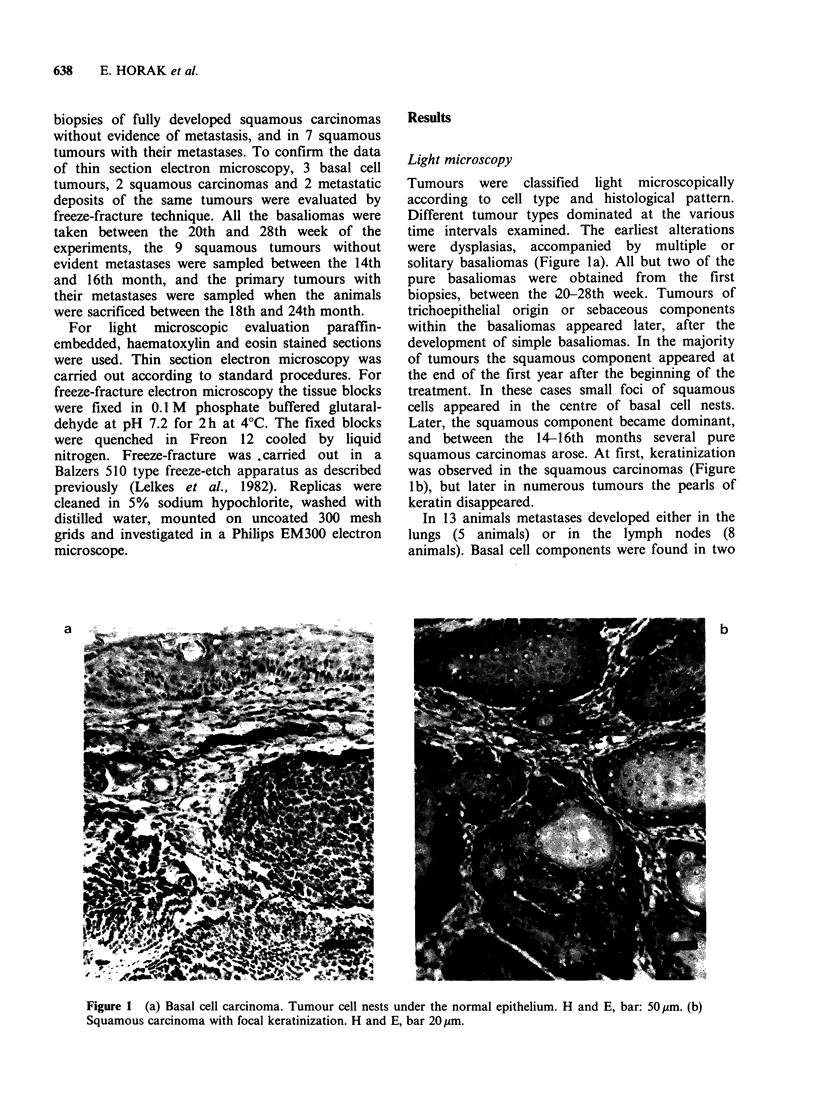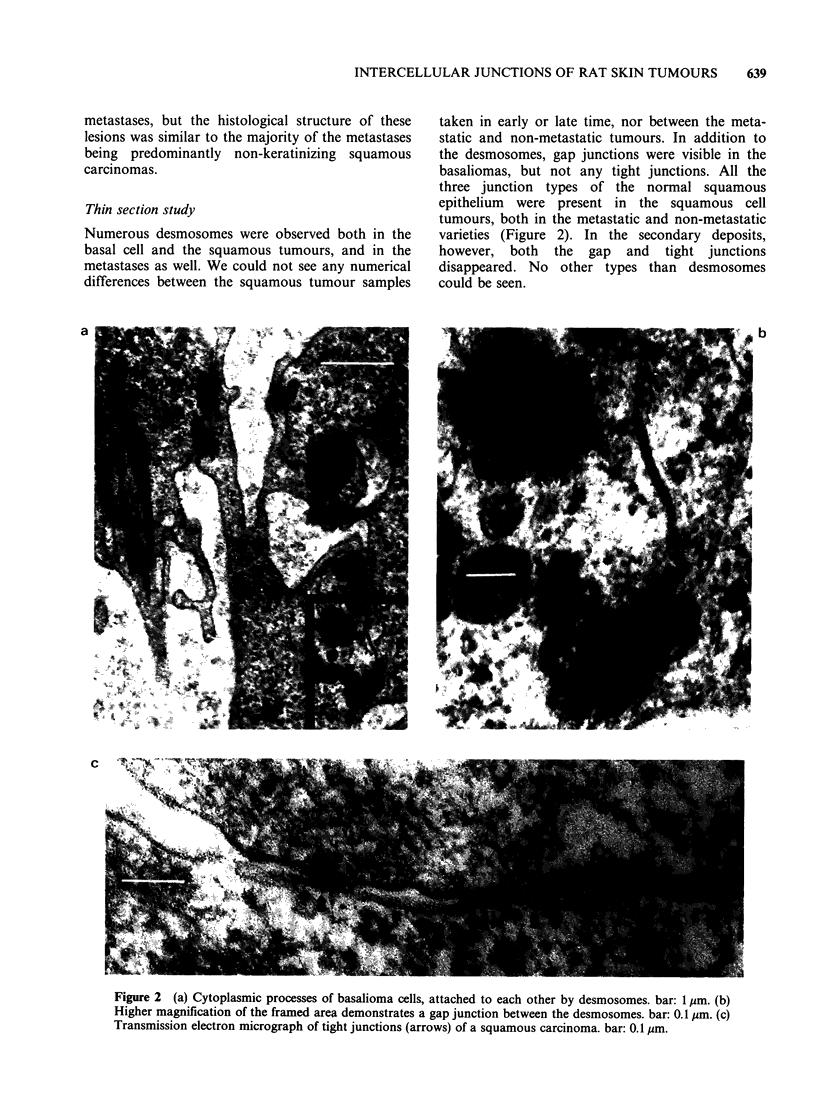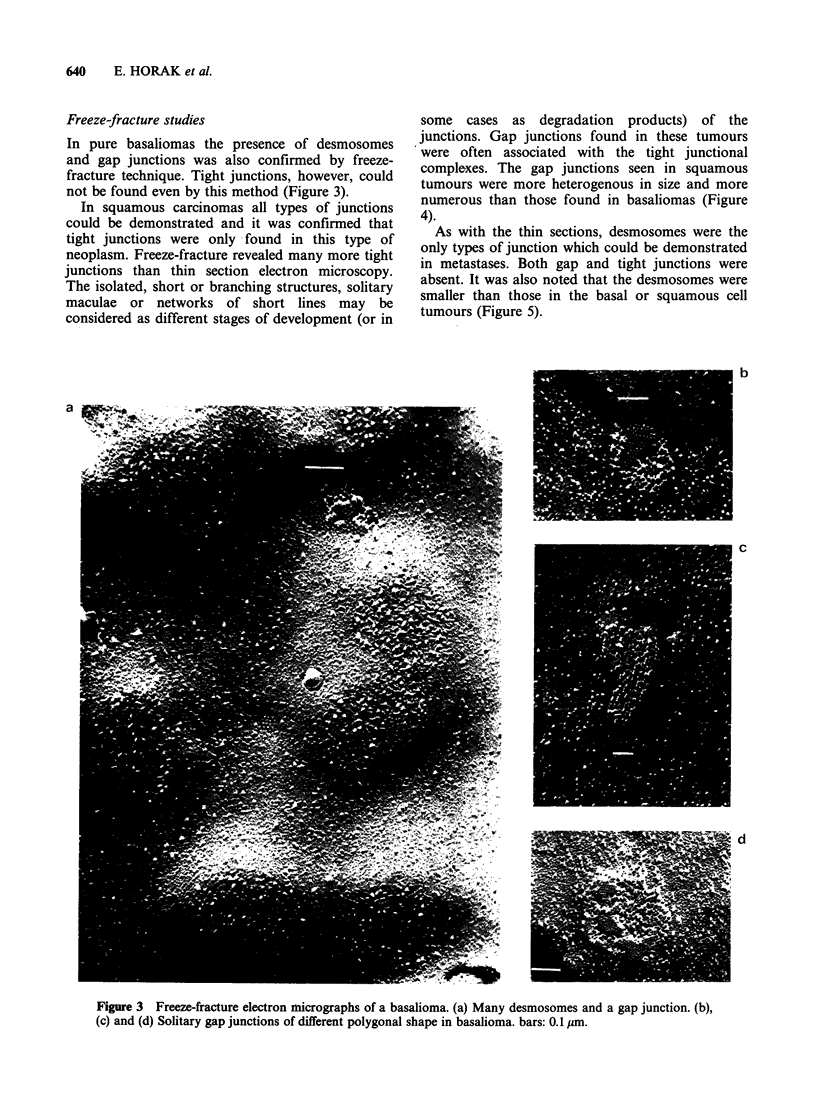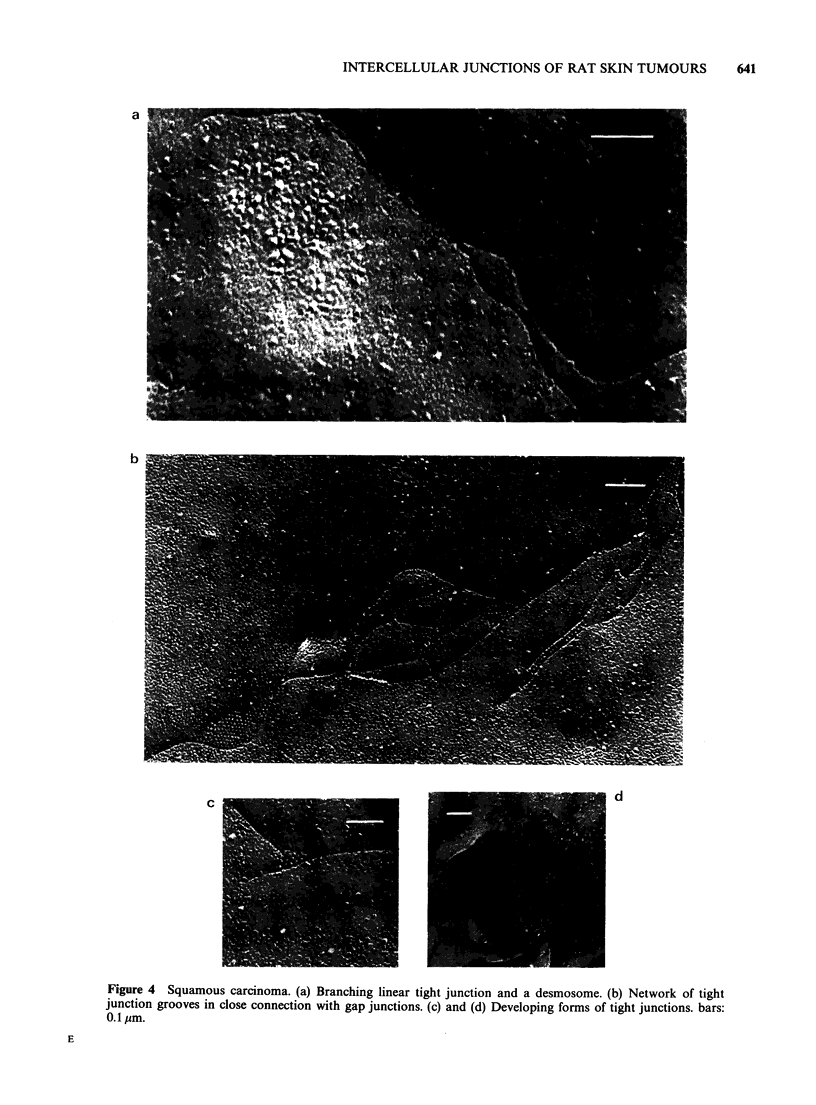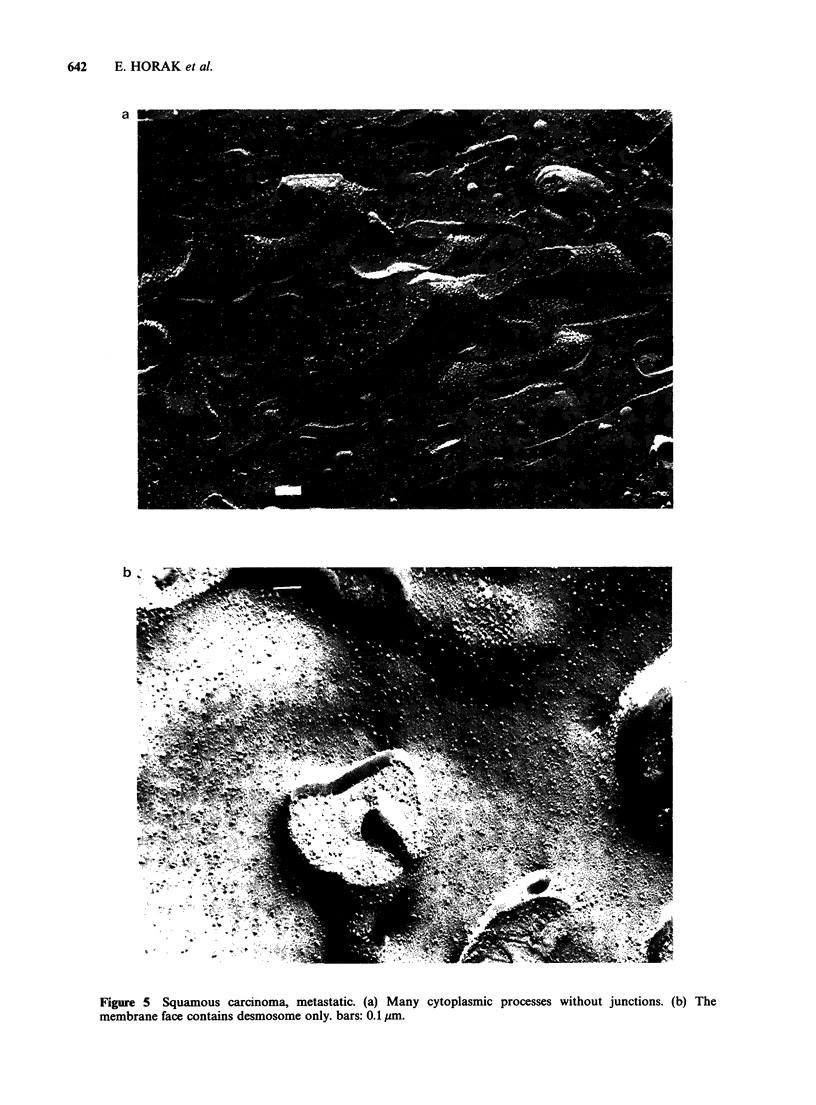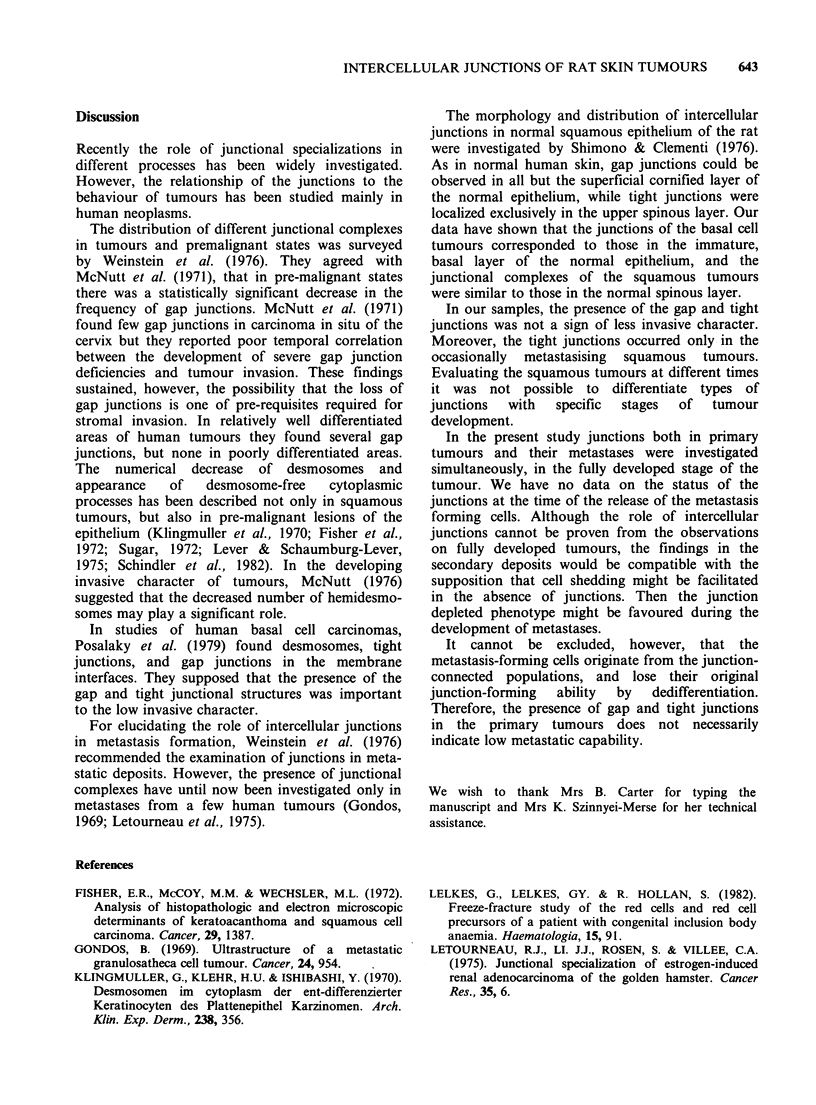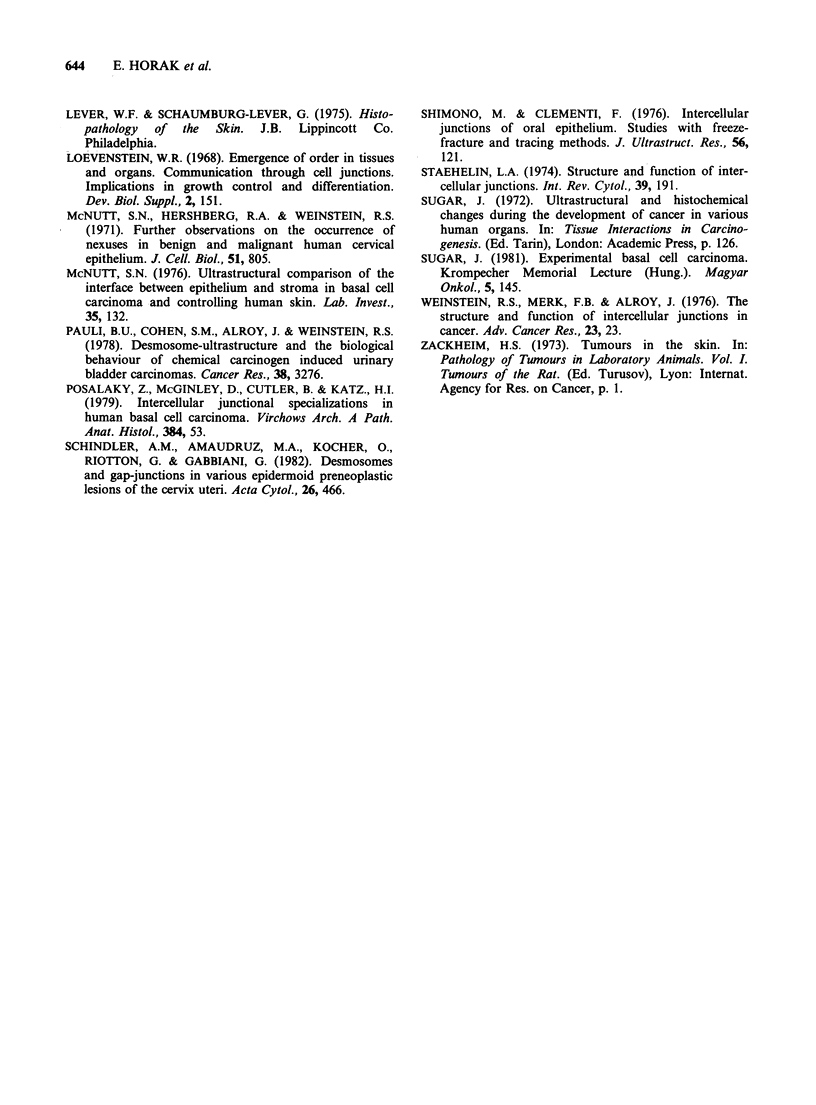# Intercellular junctions of methylcholanthrene-induced rat skin basocellular and squamous carcinomas.

**DOI:** 10.1038/bjc.1984.99

**Published:** 1984-05

**Authors:** E. Horak, G. Lelkes, J. Sugar

## Abstract

**Images:**


*Br. J. Cancer* (1984), **49**, 637–644

### Intercellular junctions of methylcholanthrene-induced rat skin basocellular and squamous carcinomas

E. Horak[1], G. Lelkes[2] & J. Sugar[1]

[1]*Research Institute of Oncopathology, National Oncological Institute,* [2]*National Institute of Haematology and Blood Transfusion, Budapest, Hungary.*

**Summary** The occurrence of different intercellular junctions in epithelial rat skin tumours induced by methylcholanthrene was investigated using thin sections and freeze-fracture replicas examined by electron microscopy. Tumours which appeared first were basal cell carcinomas. Later, different tumours of hair follicle and of sebaceous gland origin were formed. Finally, in the majority of tumours a squamous component evolved. Metastases developed from the squamous carcinomas exclusively.

Desmosomes and gap junctions were detected in basal cell carcinomas whereas, in squamous carcinomas, tight junctions were also seen. While all three types of junction were found in the primary squamous tumours, the tumour metastases in lymph nodes and lungs contained only desmosomes.

In earlier studies of the invasiveness and metastatic capacity of various tumours particular attention has been paid to the investigation of junctional complexes. From these it has been found that the occurrence and development of different junctions appears to be related to the degree of tumour differentiation (Loevenstein, 1968; McNutt *et al.*, 1971; Staehelin, 1974; Weinstein *et al.*, 1976). Moreover, evaluation of the types of junctional complexes present in different tumours may also add to our knowledge about metastasis formation (McNutt *et al.*, 1971; 1976; Weinstein, 1976; Pauli *et al.*, 1978).

The locally invasive human basal cell carcinoma grows slowly and exceptionally rarely forms metastases. The occurrence and structure of the junctional complexes well characterizes the different phases of invasive growth of these tumours (Posalaky *et al.*, 1979).

The pure forms of rat skin basocellular carcinomas are similar to the human examples in their morphological appearance and natural history. However, the frequent pilosebaceous differentiation and the simultaneously appearing squamous component in some experimentally induced skin neoplasms show that considerable differences exist (Zackheim, 1973; Sugar, 1981). The pure basal cell tumours never develop metastases, while in some cases of the later appearing squamous tumours, metastases appear in the regional lymph nodes or in the lungs.

Correspondence: E. Horak, Nuffield Department of Pathology, University of Oxford, John Radcliffe Hospital, Headington, Oxford OX3 9DU, UK.

Received 6 January 1984; accepted 31 January 1984.

The objective of the present study was to ascertain whether the presence or absence of certain types of intercellular junctions could be correlated with the invasive or metastatic behaviour of different histological types of skin carcinomas. The junctional complexes of experimentally induced rat skin tumours and their metastases were examined by thin section electron microscopy as well as by freeze-fracture studies.

### Materials and methods

Skin tumours were induced in male Wistar rats by topical application of 3% methylcholanthrene, dissolved in acetone. The back skin of the animals was painted three times weekly for a year. The first lesions appeared in the sixth month after the beginning of treatment, as previously described by Sugar (1981). In this investigation we studied the sequential changes in the skin tumours of 96 animals by light microscopic investigation of 147 biopsies from the primary lesions and in 13 samples from the metastases. The first biopsies were taken when the primary tumours were 3–5 mm in diameter (between the 6th–8th month after the beginning of the treatment) and the biopsies were repeated in the 9–17th month when the tumours were fully developed (1.5–2.5 cm in diameter). The animals were sacrificed if the tumour exceeded this size or was ulcerated. Pulmonary or lymph node metastases developed in 13/23 animals surviving $>18$ months from the beginning of the experiment.

From the biopsies, 61 tumours were suitable for thin section electron microscopy. Among them the intercellular junctions were evaluated only in the pure forms of the tumours, i.e. in 8 basaliomas, 9

© The Macmillan Press Ltd., 1984

biopsies of fully developed squamous carcinomas without evidence of metastasis, and in 7 squamous tumours with their metastases. To confirm the data of thin section electron microscopy, 3 basal cell tumours, 2 squamous carcinomas and 2 metastatic deposits of the same tumours were evaluated by freeze-fracture technique. All the basaliomas were taken between the 20th and 28th week of the experiments, the 9 squamous tumours without evident metastases were sampled between the 14th and 16th month, and the primary tumours with their metastases were sampled when the animals were sacrificed between the 18th and 24th month.

For light microscopic evaluation paraffin-embedded, haematoxylin and eosin stained sections were used. Thin section electron microscopy was carried out according to standard procedures. For freeze-fracture electron microscopy the tissue blocks were fixed in 0.1 M phosphate buffered glutaraldehyde at pH 7.2 for 2h at 4°C. The fixed blocks were quenched in Freon 12 cooled by liquid nitrogen. Freeze-fracture was carried out in a Balzers 510 type freeze-etch apparatus as described previously (Lelkes *et al.*, 1982). Replicas were cleaned in 5% sodium hypochlorite, washed with distilled water, mounted on uncoated 300 mesh grids and investigated in a Philips EM300 electron microscope.

### Results

### *Light microscopy*

Tumours were classified light microscopically according to cell type and histological pattern. Different tumour types dominated at the various time intervals examined. The earliest alterations were dysplasias, accompanied by multiple or solitary basaliomas (Figure 1a). All but two of the pure basaliomas were obtained from the first biopsies, between the 20–28th week. Tumours of trichoepithelial origin or sebaceous components within the basaliomas appeared later, after the development of simple basaliomas. In the majority of tumours the squamous component appeared at the end of the first year after the beginning of the treatment. In these cases small foci of squamous cells appeared in the centre of basal cell nests. Later, the squamous component became dominant, and between the 14–16th months several pure squamous carcinomas arose. At first, keratinization was observed in the squamous carcinomas (Figure 1b), but later in numerous tumours the pearls of keratin disappeared.

In 13 animals metastases developed either in the lungs (5 animals) or in the lymph nodes (8 animals). Basal cell components were found in two

**Figure 1** (a) Basal cell carcinoma. Tumour cell nests under the normal epithelium. H and E, bar: 50 $\mu$m. (b) Squamous carcinoma with focal keratinization. H and E, bar 20 $\mu$m.

metastases, but the histological structure of these lesions was similar to the majority of the metastases being predominantly non-keratinizing squamous carcinomas.

*Thin section study*

Numerous desmosomes were observed both in the basal cell and the squamous tumours, and in the metastases as well. We could not see any numerical differences between the squamous tumour samples taken in early or late time, nor between the metastatic and non-metastatic tumours. In addition to the desmosomes, gap junctions were visible in the basaliomas, but not any tight junctions. All the three junction types of the normal squamous epithelium were present in the squamous cell tumours, both in the metastatic and non-metastatic varieties (Figure 2). In the secondary deposits, however, both the gap and tight junctions disappeared. No other types than desmosomes could be seen.

**Figure 2** (a) Cytoplasmic processes of basalioma cells, attached to each other by desmosomes. bar: 1 $\mu$m. (b) Higher magnification of the framed area demonstrates a gap junction between the desmosomes. bar: 0.1 $\mu$m. (c) Transmission electron micrograph of tight junctions (arrows) of a squamous carcinoma. bar: 0.1 $\mu$m.

*Freeze-fracture studies*

In pure basaliomas the presence of desmosomes and gap junctions was also confirmed by freeze-fracture technique. Tight junctions, however, could not be found even by this method (Figure 3).

In squamous carcinomas all types of junctions could be demonstrated and it was confirmed that tight junctions were only found in this type of neoplasm. Freeze-fracture revealed many more tight junctions than thin section electron microscopy. The isolated, short or branching structures, solitary maculae or networks of short lines may be considered as different stages of development (or in some cases as degradation products) of the junctions. Gap junctions found in these tumours were often associated with the tight junctional complexes. The gap junctions seen in squamous tumours were more heterogenous in size and more numerous than those found in basaliomas (Figure 4).

As with the thin sections, desmosomes were the only types of junction which could be demonstrated in metastases. Both gap and tight junctions were absent. It was also noted that the desmosomes were smaller than those in the basal or squamous cell tumours (Figure 5).

**Figure 3** Freeze-fracture electron micrographs of a basalioma. (a) Many desmosomes and a gap junction. (b), (c) and (d) Solitary gap junctions of different polygonal shape in basalioma. bars: 0.1 $\mu$m.

**Figure 4** Squamous carcinoma. (a) Branching linear tight junction and a desmosome. (b) Network of tight junction grooves in close connection with gap junctions. (c) and (d) Developing forms of tight junctions. bars: 0.1 $\mu$m.

**Figure 5** Squamous carcinoma, metastatic. (a) Many cytoplasmic processes without junctions. (b) The membrane face contains desmosome only. bars: 0.1 $\mu$m.

### Discussion

Recently the role of junctional specializations in different processes has been widely investigated. However, the relationship of the junctions to the behaviour of tumours has been studied mainly in human neoplasms.

The distribution of different junctional complexes in tumours and premalignant states was surveyed by Weinstein *et al.* (1976). They agreed with McNutt *et al.* (1971), that in pre-malignant states there was a statistically significant decrease in the frequency of gap junctions. McNutt *et al.* (1971) found few gap junctions in carcinoma in situ of the cervix but they reported poor temporal correlation between the development of severe gap junction deficiencies and tumour invasion. These findings sustained, however, the possibility that the loss of gap junctions is one of pre-requisites required for stromal invasion. In relatively well differentiated areas of human tumours they found several gap junctions, but none in poorly differentiated areas. The numerical decrease of desmosomes and appearance of desmosome-free cytoplasmic processes has been described not only in squamous tumours, but also in pre-malignant lesions of the epithelium (Klingmuller *et al.*, 1970; Fisher *et al.*, 1972; Sugar, 1972; Lever & Schaumburg-Lever, 1975; Schindler *et al.*, 1982). In the developing invasive character of tumours, McNutt (1976) suggested that the decreased number of hemidesmosomes may play a significant role.

In studies of human basal cell carcinomas, Posalaky *et al.* (1979) found desmosomes, tight junctions, and gap junctions in the membrane interfaces. They supposed that the presence of the gap and tight junctional structures was important to the low invasive character.

For elucidating the role of intercellular junctions in metastasis formation, Weinstein *et al.* (1976) recommended the examination of junctions in metastatic deposits. However, the presence of junctional complexes have until now been investigated only in metastases from a few human tumours (Gondos, 1969; Letourneau *et al.*, 1975).

The morphology and distribution of intercellular junctions in normal squamous epithelium of the rat were investigated by Shimono & Clementi (1976). As in normal human skin, gap junctions could be observed in all but the superficial cornified layer of the normal epithelium, while tight junctions were localized exclusively in the upper spinous layer. Our data have shown that the junctions of the basal cell tumours corresponded to those in the immature, basal layer of the normal epithelium, and the junctional complexes of the squamous tumours were similar to those in the normal spinous layer.

In our samples, the presence of the gap and tight junctions was not a sign of less invasive character. Moreover, the tight junctions occurred only in the occasionally metastasising squamous tumours. Evaluating the squamous tumours at different times it was not possible to differentiate types of junctions with specific stages of tumour development.

In the present study junctions both in primary tumours and their metastases were investigated simultaneously, in the fully developed stage of the tumour. We have no data on the status of the junctions at the time of the release of the metastasis forming cells. Although the role of intercellular junctions cannot be proven from the observations on fully developed tumours, the findings in the secondary deposits would be compatible with the supposition that cell shedding might be facilitated in the absence of junctions. Then the junction depleted phenotype might be favoured during the development of metastases.

It cannot be excluded, however, that the metastasis-forming cells originate from the junction-connected populations, and lose their original junction-forming ability by dedifferentiation. Therefore, the presence of gap and tight junctions in the primary tumours does not necessarily indicate low metastatic capability.

We wish to thank Mrs B. Carter for typing the manuscript and Mrs K. Szinnyei-Merse for her technical assistance.

### References

FISHER, E.R., McCOY, M.M. & WECHSLER, M.L. (1972). Analysis of histopathologic and electron microscopic determinants of keratoacanthoma and squamous cell carcinoma. *Cancer*, **29**, 1387.

GONDOS, B. (1969). Ultrastructure of a metastatic granulosatheca cell tumour. *Cancer*, **24**, 954.

KLINGMULLER, G., KLEHR, H.U. & ISHIBASHI, Y. (1970). Desmosomen im cytoplasm der ent-differenzierter Keratinocyten des Plattenepithel Karzinomen. *Arch. Klin. Exp. Derm.*, **238**, 356.

LELKES, G., LELKES, GY. & R. HOLLAN, S. (1982). Freeze-fracture study of the red cells and red cell precursors of a patient with congenital inclusion body anaemia. *Haematologia*, **15**, 91.

LETOURNEAU, R.J., LI. J.J., ROSEN, S. & VILLEE, C.A. (1975). Junctional specialization of estrogen-induced renal adenocarcinoma of the golden hamster. *Cancer Res.*, **35**, 6.

LEVER, W.F. & SCHAUMBURG-LEVER, G. (1975). *Histopathology of the Skin.* J.B. Lippincott Co. Philadelphia.

LOEVENSTEIN, W.R. (1968). Emergence of order in tissues and organs. Communication through cell junctions. Implications in growth control and differentiation. *Dev. Biol. Suppl.*, **2**, 151.

McNUTT, S.N., HERSHBERG, R.A. & WEINSTEIN, R.S. (1971). Further observations on the occurrence of nexuses in benign and malignant human cervical epithelium. *J. Cell. Biol.*, **51**, 805.

McNUTT, S.N. (1976). Ultrastructural comparison of the interface between epithelium and stroma in basal cell carcinoma and controlling human skin. *Lab. Invest.*, **35**, 132.

PAULI, B.U., COHEN, S.M., ALROY, J. & WEINSTEIN, R.S. (1978). Desmosome-ultrastructure and the biological behaviour of chemical carcinogen induced urinary bladder carcinomas. *Cancer Res.*, **38**, 3276.

POSALAKY, Z., McGINLEY, D., CUTLER, B. & KATZ., H.I. (1979). Intercellular junctional specializations in human basal cell carcinoma. *Virchows Arch. A Path. Anat. Histol.*, **384**, 53.

SCHINDLER, A.M., AMAUDRUZ, M.A., KOCHER, O., RIOTTON, G. & GABBIANI, G. (1982). Desmosomes and gap-junctions in various epidermoid preneoplastic lesions of the cervix uteri. *Acta Cytol.*, **26**, 466.

SHIMONO, M. & CLEMENTI, F. (1976). Intercellular junctions of oral epithelium. Studies with freeze-fracture and tracing methods. *J. Ultrastruct. Res.*, **56**, 121.

STAEHELIN, L.A. (1974). Structure and function of intercellular junctions. *Int. Rev. Cytol.*, **39**, 191.

SUGAR, J. (1972). Ultrastructural and histochemical changes during the development of cancer in various human organs. In: *Tissue Interactions in Carcinogenesis*. (Ed. Tarin), London: Academic Press, p. 126.

SUGAR, J. (1981). Experimental basal cell carcinoma. Krompecher Memorial Lecture (Hung.). *Magyar Onkol.*, **5**, 145.

WEINSTEIN, R.S., MERK, F.B. & ALROY, J. (1976). The structure and function of intercellular junctions in cancer. *Adv. Cancer Res.*, **23**, 23.

ZACKHEIM, H.S. (1973). Tumours in the skin. In: *Pathology of Tumours in Laboratory Animals. Vol. I. Tumours of the Rat.* (Ed. Turusov), Lyon: Internat. Agency for Res. on Cancer, p. 1.